# Terrie Fox Wetle Award (2020): The Role of Home-Delivered Meals Programs in Improving Health and Promoting Community Independence for Older Adults

**DOI:** 10.1093/geroni/igab046.1491

**Published:** 2021-12-17

**Authors:** Kali Thomas

**Affiliations:** Brown University, Brown University/Providence, Rhode Island, United States

## Abstract

Dr. Terrie “Fox” Wetle is internationally recognized as a leader who conducts and advocates for multi-disciplinary and multi-method investigations centered on aging, public health and health care with direct implications for shaping policy and practice. This award lecture, given in Dr. Wetle’s name, will be presented by the 2020 award recipient, Kali Thomas, PhD. Dr. Thomas will present a line of multi-disciplinary and multi-method research focused on the impact of home-delivered meals as it relates to the health outcomes of homebound, food insecure older adults. Findings will include results from observational and intervention studies conducted at both the local and national levels. Examples of how this evidence has influenced policy and practice, including greater integration with healthcare, will be provided. The lecture will conclude with discussion about future opportunities for collaboration with community partners to measure and understand the impact of these vital social services on the lives of older adults.

